# Benchmark of the PenRed Monte Carlo framework for HDR brachytherapy

**DOI:** 10.1016/j.zemedi.2022.11.002

**Published:** 2022-12-09

**Authors:** Sandra Oliver, Vicent Giménez-Alventosa, Francisco Berumen, Vicente Gimenez, Luc Beaulieu, Facundo Ballester, Javier Vijande

**Affiliations:** a Instituto de Seguridad Industrial, Radiofísica y Medioambiental (ISIRYM), Universitat Politècnica de València, Camí de Vera s/n, 46022 València, Spain; b Escuela de Ciencias, Ingeniería y Diseño, Universidad Europea de Valencia, Paseo de la Alameda 7, 46010 València, Spain; c Instituto de Instrumentación para Imagen Molecular (I3M), Centro mixto CSIC - Universitat Politècnica de València, Camí de Vera s/n, 46022 València, Spain; d Département de Radio-Oncologie et Axe oncologie du Centre de recherche du CHU de Québec, CHU de Québec, Québec, QC, Canada; e Département de Physique, de Génie Physique et d’Optique et Centre de Recherche sur le Cancer, Université Laval, Québec, QC, Canada; f Departament de Física Teórica and IFIC, Universitat de València-CSIC, Dr. Moliner, 50, 46100 Burjassot, València, Spain; g Departamento de Física Atómica, Molecular y Nuclear. IRIMED, IIS-La Fe-Universitat de Valencia, 46100 Burjassot, Spain; h Instituto de Física Corpuscular, IFIC (UV-CSIC), 46100 Burjassot, Spain

**Keywords:** Monte Carlo, PenRed, Brachytherapy, DICOM, Medical physics

## Abstract

**Purpose:**

The purpose of this study is to validate the PenRed Monte Carlo framework for clinical applications in brachytherapy. PenRed is a C++ version of Penelope Monte Carlo code with additional tallies and utilities.

**Methods and materials:**

Six benchmarking scenarios are explored to validate the use of PenRed and its improved bachytherapy-oriented capabilities for HDR brachytherapy. A new tally allowing the evaluation of collisional kerma for any material using the track length kerma estimator and the possibility to obtain the seed positions, weights and directions processing directly the DICOM file are now implemented in the PenRed distribution. The four non-clinical test cases developed by the Joint AAPM-ESTRO-ABG-ABS WG-DCAB were evaluated by comparing local and global absorbed dose differences with respect to established reference datasets. A prostate and a palliative lung cases, were also studied. For them, absorbed dose ratios, global absorbed dose differences, and cumulative dose-volume histograms were obtained and discussed.

**Results:**

The air-kerma strength and the dose rate constant corresponding to the two sources agree with the reference datatests within 0.3% ($S_{k}$) and 0.1% (Λ). With respect to the first three WG-DCAB test cases, more than 99.8% of the voxels present local (global) differences within ±1%(±0.1%) of the reference datasets. For test Case 4 reference dataset, more than 94.9%(97.5%) of voxels show an agreement within ±1%(±0.1%), better than similar benchmarking calculations in the literature. The track length kerma estimator scorer implemented increases the numerical efficiency of brachytherapy calculations two orders of magnitude, while the specific brachytherapy source allows the user to avoid the use of error-prone intermediate steps to translate the DICOM information into the simulation. In both clinical cases, only minor absorbed dose differences arise in the low-dose isodoses. 99.8% and 100% of the voxels have a global absorbed dose difference ratio within ±0.2% for the prostate and lung cases, respectively. The role played by the different segmentation and composition material in the bone structures was discussed, obtaining negligible absorbed dose differences. Dose-volume histograms were in agreement with the reference data.

**Conclusions:**

PenRed incorporates new tallies and utilities and has been validated for its use for detailed and precise high-dose-rate brachytherapy simulations.

## 1 Introduction

Brachytherapy (BT) is a well known therapeutic technique for the treatment of tumour lesions that consists on the placement of an encapsulated radioactive source close to, in contact with, or within a tumor to irradiate such oncological lesion. It is commonly used as an effective treatment modality for cervical, prostate, breast, and skin cancer. It has also been shown to be effective in treating tumors in the brain, head and neck region, eye, trachea and bronchi, digestive system, urinary tract, the female reproductive tract, and other soft tissues [1]. A typical example would be the use of brachytherapy in combination with external radiotherapy for patients diagnosed with cervical cancer, where an increase of about 25% has been reported for the rates of primary complete remission and 5-year cancer-specific survival [2].

The complexity and precision of brachytherapy treatments have increased drastically in recent years with the development of novel techniques like electronic brachytherapy [3], intensity modulated brachytherapy [4], directional sources [5], 3D printing [6], in vivo dosimetry [7], optimization techniques [8], among others. However, at the same time, dosimetry in clinical practice is still based on the TG-43 protocol, proposed by the American Association of Physicists in Medicine (AAPM) in the early 90s [9], [10], [11]. This formalism, although representing at the time a great improvement over the existing methods for planning brachytherapy treatments, is based on a set of precalculated data tables assuming a single source located within an infinite water volume. Model-based dose calculation algorithms (MBDCAs) are now beginning to be used in clinical practice incorporating not only the patient anatomical particularities, but also material and geometrical considerations from both applicators and shielding [12]. Examples of MBDCAs commercially available for clinical practice are collapsed–cone point kernel superposition methods, ACE (Elekta Brachy, Veenendaal, The Netherlands) and grid-based linear Boltzmann solvers, AcurosBV (Varian Medical System, Palo Alto, CA).

Although the improvement offered by existing MBDCAs is evident, not all of them are able to describe the dose deposition in brachytherapy with the same level of precision as Monte Carlo (MC) methods [13]. Due to its accuracy in the modeling of the physical process involved, MC is considered nowadays the gold standard for dose calculations in brachytherapy. There are several MC suites and toolkits available in medical physics. Among those, some have been commonly used in brachytherapy: BrachyDose [14], egs_brachy [15], ALGEBRA [16], RapidBrachyMCTPS [17], MCNP [18], TOPAS Brachy [19], [20], and Penelope [21]. PenRed [22] is a new addition to that list. PenRed expands the MC suite Penelope incorporating fully share and distributed memory parallelism. It also includes tallies and functionalities ideally suited for its application in the study of brachytherapy dosimetry.

The purpose of this study is to validate PenRed for high-dose rate (HDR) clinical applications in brachytherapy. To do so, a similar procedure as the one recommended by TG-186 and the AAPM-ESTRO-ABG-ABS Working Group on Model Based Dose Calculation Algorithms (WG-DCAB) has been followed. First, the four test cases released by the WG-DCAB [23], [24], [25] available at the IROC Houston Brachytherapy Source Registry [26] have been evaluated. Secondly, two different clinical cases were studied and compared against previously validated reference datasets [20].

## 2 Materials and methods

### 2.1 Monte Carlo code

The MC code PenRed v.1.5.2 [22] comes up with a translation to C++ of the original Penelope package [21], which was reorganised with a modular and extensible structure using an Object-Oriented programming model, providing a more flexible code. As Penelope, PenRed is designed to simulate the transport of electrons, photons and positrons, within the energy range from 50 eV to 1 GeV, in arbitrary materials, including all the Penelope physics implementation. The main interactions of particle transport are described by means of the corresponding differential cross sections (DCSs). The photoelectric effect is simulated through DCSs calculated with the program PHOTACS by using conventional first-order perturbation theory [27], [28]. For the Compton interactions, the DCSs are calculated from the relativistic impulse approximation with analytical one-electron Compton profiles, which takes into account both binding effects and Doppler broadening [29]. The Rayleigh scattering is simulated by means of DCSs calculated using non-relativistic perturbation theory in the Born approximation, in which the form and effective anomalous scattering factors [30] are obtained from EPDL97 [31]. For the case of emission of characteristics X-ray and Auger electrons, they are simulated using the transition probabilities of the vacancies towards outer shells [32]. The total cross sections for the pair and triplet production are obtained from the XCOM program [33].

With respect to the interactions produced by charged particles, the elastic scattering is simulated using numerical DCSs obtained with ELSEPA [34], [35], by using the relativistic Dirac partial-wave method, while the inelastic collisions are simulated using the generalized oscillator strength model [36], [37]. The bremsstrahlung photon emission is sampled from numerical energy-loss spectra derived from the scaled cross-section tables of Seltzer and Berger [38], [39]. The angular distribution of these emitted photons is simulated using an analytical expression [40] whose parameters are calculated with the BREMS program [41]. Finally, the positron annihilation is simulated by using the Heitler DCSs [42] for in-flight two-photon annihilation with free electrons at rest.

PenRed is an open-source project available in github^1^ designed to extend the capabilities of the original Penelope suite. Briefly, with the aim to improve the simulation efficiency, PenRed implements parallelism at two levels, multithreading and multiprocess based on the Message Passing Interface (MPI) standard, therefore being specifically designed and optimized for massively parallel infrastructures. With respect to the detailed simulation of clinical cases, besides optimizing the original geometry library, PenRed goes beyond the implementation of voxelized geometries in Penelope by allowing the user to incorporate directly DICOM datasets. Notice that PenRed DICOM capabilities are vendor independent since it follows the DICOM standard specifications. This feature allows to process DICOM files from different image modalities and also to process the information from RTSTRUCT and RTPLAN. In the latter case, two techniques previously available in the PenRed code have been combined to perform the segmentation: intensity-range technique combined with the contour information extracted from the RTSTRUCT. A detailed description of these techniques can be found in the PenRed user manual github^2^. In addition, PenRed uses the contour information to define the scoring volumes. To be able to simulate the particle emission in brachytherapy treatments, we have implemented a brachytherapy source which uses the PenRed capability to process automatically the RTPLAN file and extract the seed information. A description of this source is already included in the PenRed manual.

With respect to the modular structure of the code, PenRed allows the user to easily incorporate new custom components, such as particle sources or tallies, to extend the code for specific applications. For instance, the linear track-length kerma estimator (TLKE) [43] is a variance reduction technique widely used in BT. The TLKE allows the user to score the collisional kerma as a surrogate of the absorbed dose.

The TLKE defined as:

(1) $$D\approx K_{\mathrm{coll}}=\int \Phi_{E}E{\left(\frac{\mu_{\mathrm{en}}}{\rho }\right)}_{E}\mathrm{dE}=\frac{1}{V}\underset{i}{\sum }l_{i}E_{i}{\left(\frac{\mu_{\mathrm{en}}}{\rho }\right)}_{E_{i}},$$

where *D* is the absorbed dose, $\Phi_{E}$ the photon fluence, $\mu_{\mathrm{en}}/\rho$ the mass-energy absorption coefficient for the voxel material, *V* the voxel volume, and $l_{i}$ the distance travelled by a photon with energy $E_{i}$, takes advantage of the particle fluence and total photon path length per unit volume equivalence [44]. Therefore, each photon contributes to all scoring volumes traversed instead of only the one in which a particular interaction occurs. This technique allows to reduce the variance faster than the usual dose scoring methods.

However, to approximate absorbed dose as collisional kerma, charged particle equilibrium must be fulfilled. By assuming charge particle equilibrium, secondary electrons are not tracked. Therefore, this expression will only be valid in those cases where their energy is deposited locally [43]. In the particular case of ${}^{192}$Ir-based brachytherapy dosimetry this is always the case with the exception of those locations in the source vicinity or very close to an interface. As it is shown in [45], absorbed dose may be safely approximated as collisional kerma within 1 % at distances greater than 2 mm from the source for the case of Ir-192. That is not the case for ${}^{60}$Co-based brachytherapy, where dose should be scored up to a minimum of 7 mm from any source.

For this purpose, the authors have implemented a TLKE tally in PenRed expanding its applicability. This tally calculates the collisional kerma as a surrogate of the absorbed dose for each contoured structure of a DICOM-RT data set. To help the user in analysing clinical cases, cumulative dose-volume histograms (DVHs) are automatically reported by this tally when contoured CT image volumes exist. More details of the implemented DICOM_KERMA_TRACK_LENGTH tally can be found in the usage guide included in the PenRed v.1.5.2 package. Therefore, in all the following simulations, collisional kerma will be scored.

Additionally, a new brachytherapy-specific capability was implemented for the PenRed distribution consisting on a new source tally named brachy_source. It allows to set the position and direction of the generated particles according to the information provided in the corresponding DICOM-RT file without intermediate steps. brachy_source uses catheter information to provide the actual source orientation within the patient anatomy. Moreover, the sampling in each position takes into account the dwell times. Details on its implementation and parameters can be found in the PenRed documentation.

Since no electron transport was considered the parameters required for the mixed (class-II) algorithm, namely C1,C2,WCC,WCR and *DSMAX*, are irrelevant. Regarding photons, as it is shown in Table 1, the absorption energy was taken at 1 keV The main characteristics and parameters used for the Monte Carlo simulations are summarized in Table 1, which follows the recommendations of the Task Group 268 of the AAPM[46].

**Table 1:** Summary of the main characteristics of the Monte Carlo simulations used in this work.

Item	Description	References
Code	PenRed v.1.5.2	[22]
Validation	Previously validated	[22]
Timing	Intel(R) Core(TM) i9-9900 K CPU @3.60 GHz	
	Source validation: 55 CPU hours and 1×10^10^ histories	
	Water cube phantom tests: 1134 CPU hours and 5×10^10^ histories	
	Intel(R) Core(TM) i9-10900 K CPU @ 3.70 GHz	
	Clinical source PSF: 5 CPU minutes and 5×10^7^ histories	
	Clinical cases: 35 CPU hours and 5×10^10^ histories	
Source description	Generic WG-DCAB Ir-192 source	[23]
	MicroSelectron–HDR-v2 Ir-192 source	[47]
Cross-sections	Photoelectric from PHOTACS	[27], [28]
	Compton from relativistic impulse approximation	[29]
	Rayleigh scattering using non-relativistic perturbation theory	[30]
	X-ray and Auger electrons using transition probabilities	[32]
	Elastic scattering using numerical DCSs	[34], [35]
	Bremsstrahlung photon emission using tables of Seltzer and Berger	[38], [39]
	Positron annihilation using the Heitler DCSs	[42]
Transport parameters	Photon cut-off = 1 keV in all materials	
	No electron transport was considered (C1,C2,WCC,WCR and *DSMAX* are irrelevant)	
Variance reduction tools	Water cube phantom tests: no VR used	
	Clinical tests: splitting particles used for PSF source	[21]
Scored quantities	Water cube phantom tests: kerma in water	
	Clinical tests: kerma in organs of interest	
Statistical uncertainties	⩽0.25%	
Postprocessing	None	

### 2.2 WG-DCAB and microSelectron–HDR v2 Ir-192 sources

In this work, two brachytherapy sources have been used: the generic WG-DCAB Ir-192 source [23] and the microSelectron–HDR v2 Ir-192 source [47]. Both sources were modeled for MC simulations and their air-kerma strength ($S_{k}$) and dose-rate constant (Λ) were calculated following the AAPM recommendations [9]. To do so, the source was first positioned in vacuum except for a cylindrical air cell with a width of 0.1 cm and height of 0.1 cm located at *r* = 10 cm where the air-collision kerma was scored to obtain the $S_{k}$. Then, the source was located at the center of a water sphere with a radius of 40 cm to measure the collision kerma in a cylindrical voxel of *r* = 0.5 mm thickness and 1 mm height located at 1 cm from the source center along the transverse axis.

### 2.3 Benchmarking simulations

As discussed above, two different sets of tests will be performed following the procedure outlined by the WG-DCAB. First, four non-clinical test cases released by such WG have been evaluated. Then, two different clinical cases were studied. In all cases the results were compared against previously validated reference datasets.

#### 2.3.1 Water cube phantom tests

The WG-DCAB has released four non-clinical test cases to benchmark MBDCAs. Those will be used here to validate PenRed for brachytherapy applications [23], [48], [20]. The geometries of these test cases consist of a single source located within a voxelized computational model of a homogeneous water cube (Test Case 1), and a water cube surrounded by an air cube for Test Cases 2 to 4. The source, the WG-DCAB Ir-192 seed, was placed as indicated in the WG-DCAB publication. For the Test Case 1, it was located in the center of a 51.1 cm side water cube. In the Test Case 2, the “source centered in water” case, the source was positioned at the center of a 20.1 cm side water cube within a 51.1 cm side air cube. Then, Test Case 3 corresponds to the “source displaced” case, which uses the same phantom described for Test Case 2 but with the generic source displaced 7 cm along the positive *x* axis. Finally, Test Case 4 was the “source centered in applicator” case, which also uses the Test Case 2 phantom but with the source centered in the generic TG-186 shielded applicator described in [48]. Although non-clinical, these test cases allow us to benchmark different scenarios that might be relevant in brachytherapy simulations. Test Case 1 is equivalent to a TG-43 dose calculation, Test Cases 2 and 3 evaluate the influence of the lack of scattering, and Test Case 4 includes the attenuation due to large *Z* materials.

For each simulated case, the TLKE was scored in a centered and cubic voxelized mesh with a side of 20.1 cm, using voxels of 1 mm^3^. The number of photon histories was set to $5\times 10^{10}$ in all cases to yield Type A uncertainties lower than 0.25 %.

After performing the PenRed simulations, absorbed dose histograms were investigated and compared against the reference datasets obtained using the MCNP MC code. Following the protocol defined by the WG-DCAB [23] three different metrics were used:

• The local dose difference ratio, defined as $\Delta D_{\mathrm{LOCAL}}=\frac{D(r)-D_{\mathrm{ref}}(r)}{D_{\mathrm{ref}(r)}}$, represents the difference between the PenRed dose (D(r)) and the reference dose ($D_{\mathrm{ref}}(r)$) at each voxel, normalized to the reference dose at the same voxel.
• The global dose difference ratio was defined as $\Delta D_{\mathrm{GLOBAL}}=\frac{D(r)-D_{\mathrm{ref}}(r)}{D_{\mathrm{ref}(\mathrm{ref})}}$ where in this case, the difference between PenRed and the reference dose is normalized to the reference dose at a clinically relevant reference voxel ($D_{\mathrm{ref}}(\mathrm{ref})$), located at 1 cm from the source center (Test Cases 1 to 3) and 1 cm from the applicator side (Test Case 4) along the negative *x* direction.
• Relative dose maps depicting $\frac{D(r)\times {\left(r-r_{s}\right)}^{2}}{D(r_{\mathrm{ref}})\times {\left(r_{\mathrm{ref}}-r_{s}\right)}^{2}}$ where $r_{s}$ is the corresponding source position for each case, i.e, at (0,0,0) cm for Cases 1, 2 and 4 and at (7, 0 0) cm for Case 3.

Voxels within the source and/or the applicator are omitted from the metrics analysis.

#### 2.3.2 Clinical tests

To evaluate the PenRed capabilities to simulate a realistic clinical brachytherapy treatment, two clinical cases were studied. These have been previously used to validate a commercial treatment planning system [49] and a MC brachytherapy toolkit [20]. First, a typical HDR prostate case was studied. This case consists of 104 CT slices with slice thickness of 2 mm and with pixel spacing of 0.371 mm × 0.371 mm. Thus, the total volume of this clinical case is 19 × 19 × 20.8 cm^3^. The CT image set includes four contoured volumes, the prostate or target, the bladder, the urethra and the rectum. Moreover, a body contour is included to enclose the rest of the CT image structures. Regarding the bone segmentation, no bone contouring was provided. In the original work [49] the cortical bone was segmented by histogram thresholding while in subsequent benchmarking works using the same case, water was assigned as bone material [20]. To turn this challenge into an opportunity, two different cases have been studied. In this way, we will not only be able to benchmark PenRed but also to estimate the degree of uncertainty such choices impose over a clinical simulation. The first scenario, without bone consideration, assigns water to the body contour, while the second, uses a cortical bone material to segment the bone structures by intensity range thresholding. The treatment of this clinical case consists of a seventeen catheters with 111 active dwell positions located covering the clinical target volume with a prescribed dose of 15 Gy in a single fraction.

The second clinical test case consists on a typical palliative endobronchial HDR brachytherapy clinical case. This technique can be used as a sole modality in non-irradiated patients or as a boost to palliative external beam radiotherapy. Two fractions of 7.5 Gy each, three fractions of 5 Gy each, or four fractions of 4 Gy each (prescribed at 1 cm from the source) should be given when HDR brachytherapy is used as a planned boost to supplement palliative external-beam radiation therapy of 30 Gy in 10 to 12 fractions, when patients have no previous history of radiation treatment to the chest[50]. The case considered consists of 136 CT slices with slice thickness of 1.5 mm and pixel spacing of 0.684 mm × 0.684 mm and a total volume of 35 × 35 × 20.4 cm^3^. In this case, the CT image set includes the lungs and bronchus contoured volumes and also a body contour. The palliative lung treatment consists of a single catheter with 19 active dwell positions going through the bronchus into the left lung. The catheter was located to deliver a dose of 5 Gy at 1 cm from the catheter wall.

For both clinical cases the material assignation was done following the scheme of Table 2 of [49], using TG-186 materials composition [12]. This material assignation simulates the worst-case-scenario since rectum for the first clinical case, and lungs and bronchus of the second, are assigned to air.

**Table 2:** Computer-time improvement factor gained by using the TLKE tally instead of the event-by-event dose tally.

Tally used	TLKE	Spatial dose
Number of histories	$5\times 10^{10}$	$5\times 10^{12}$
Uncertainty achieved (region of interest)	0.1 (%)	0.1 (%)
Total simulation time (s)	1.537 $\times 10^{5}$	1.942 $\times 10^{6}$
Time improvement factor $t_{\mathrm{spatial}}/t_{\mathrm{kerma}}$	12.64	

To simulate the clinical cases described, first, a phase space file (PSF) for the microSelectron–HDR v2 Ir-192 source model was generated with 5 × 10^7^ initial photons. To use the PSF file, a splitting factor of 10 has been specified to each particle in the PSF. However, the whole PSF has not been simulated for each dwell position. Instead, the position where is emitted each PSF splitted particle, is randomly selected via a pre-assigned weight for each dwell position. All these processes are automatically performed by the brachy_source, including the weight calculation which is done via the dwell position times stored in the DICOM fi.e..

In these clinical simulations, the dose was scored in the same way that for the water phantom tests, using the new PenRed TLKE tally. In these cases, the defined dose grid was aligned with the CT geometry, covering all the relevant clinical structures. Moreover, a 20 cm water shell is added to ensure full scatter in the DICOM outer regions.

Once the PenRed simulations have been performed, the results were compared with the corresponding reference data obtained using the ALGEBRA MC code [16], [49]. The comparisons were done using similar metrics as in the water cube phantom tests. On the one hand, the dose ratio maps PenRed/Reference were obtained. In addition, the isodose lines are superposed for each dose ratio map. On the other hand, histograms of the $\Delta D_{\mathrm{GLOBAL}}$ differences were also calculated where $D_{\mathrm{ref}}(\mathrm{ref})$ is the absorbed dose in the voxel in which prescription point is located. This point was found in the RTPLAN of each clinical case.

Finally, the cumulative dose-volume histograms are obtained for the contoured CT image volumes. DVHs metrics are also calculated for the prostate case and compared with the reference results.

## 3 Results

### 3.1 WG-DCAB and microSelectron–HDR v2 Ir-192 sources

Both air kerma strength and dose-rate constant have been obtained for the Ir-192 sources used in this work. For the generic WG-DCAB source, $S_{k}=(9.8296\pm 0.0030)\times 10^{-8}$ U Bq^−1^, and Λ=1.1099±0.0004 cGy h^−1^ U^−1^ have been obtained. For the microSelectron–HDR v2 source, the results were $S_{k}=(9.8583\pm 0.0030)\times 10^{-8}$ UBq^−1^, and Λ=1.110±0.0004 cGy h^−1^ U^−1^. Type A uncertainties are provided for a coverage factor k=2.

**Figure 1:**
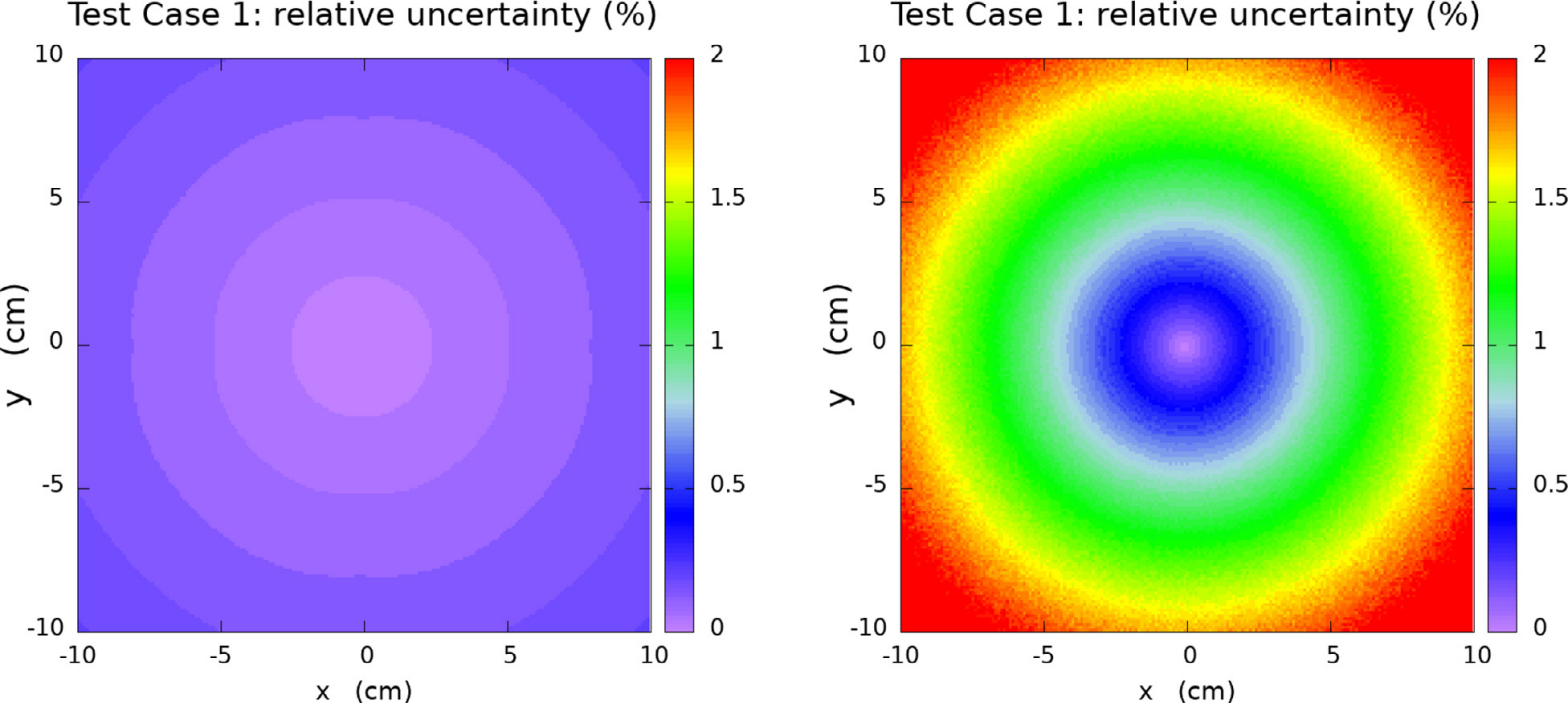
Results for relative uncertaintities of TLKE and spatial dose scorers in Test Case 1: the generic WG-DCAB Ir-192 source located in the center of 51.1 cm side water cube. Left image shows the uncertainty map for TLKE scorer and right image shows the uncertainty map for spatial absorbed dose scorer.

To illustrate the performance of the newly incorporated TLKE tally as compared with the standard analogue event-by-event dose scorer we show in Fig. 1 their relative uncertainties distributions for a fixed number of histories in the water cube Test Case 1. These results show that the relative error offered by the TLKE tally is 10 times lower than standard dose scorers. Since the Type A uncertainties decrease as $1/\sqrt{N}$, where *N* is the number of histories, to achieve the same uncertainty for Test Case 1 by means of the event-by-event dose scorer as using the TLKE tally, the number of histories will would be increased by a factor of 100. However, the TLKE algorithm requires more computational time because the track length must be scored. Therefore, the ratio in computation time between the two algorithms is not expected to be a factor 100. To determine the real improvement factor, in this case and for our hardware, we have performed the simulation with the TLKE with $5\times 10^{10}$ histories and with $5\times 10^{12}$ for the spatial dose event-by-event algorithm. The simulations have been executed in an Intel(R) Core(TM) i9-10900 K CPU @ 3.70 GHz. The resulting computation time is shown in Table 2.

### 3.2 Water cube phantom tests

**Figure 2:**
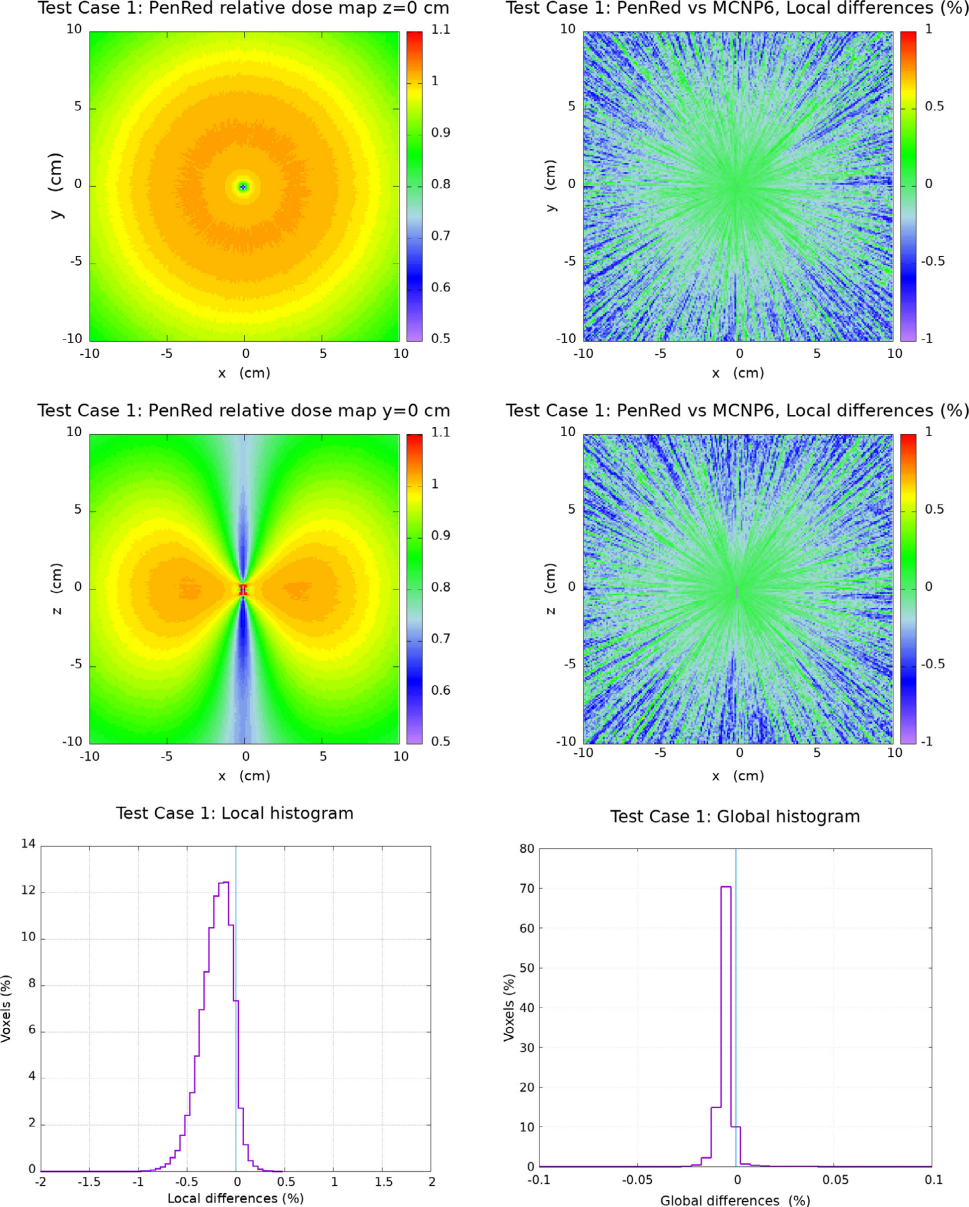
Results for Test Case 1: the generic WG-DCAB Ir-192 source located in the center of 51.1 cm side water cube. Top and middle left images show the PenRed relative dose map for z=0 and y=0 respectively. Top and middle right images show the local differences ratio $\Delta D_{\mathrm{LOCAL}}$ (%) for z=0 and y=0 respectively. Finally, bottom images show the histogram of $\Delta D_{\mathrm{LOCAL}}$ (%) (left) and $\Delta D_{\mathrm{GLOBAL}}$ (%) (right).

**Figure 3:**
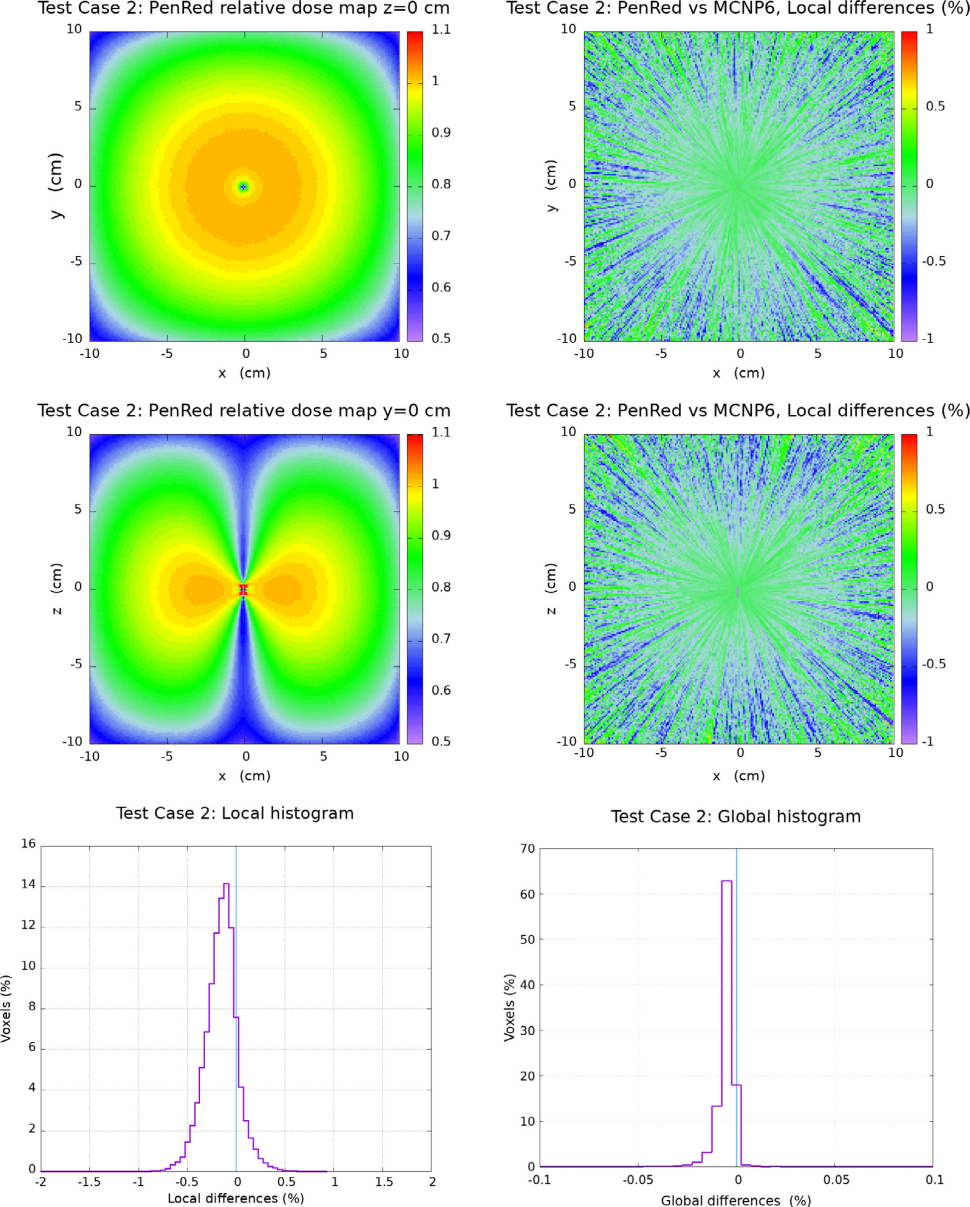
Results for Test Case 2: the “source centered in water”, the 20.1 cm side water cube is located in the center of a 51.1 cm side air cube. Top and middle left images show the PenRed relative dose map for z=0 and y=0 respectively. Top and middle right images show the local differences ratio $\Delta D_{\mathrm{LOCAL}}$ (%) for z=0 and y=0 respectively. Finally, bottom images show the histogram of $\Delta D_{\mathrm{LOCAL}}$ (%) (left) and $\Delta D_{\mathrm{GLOBAL}}$ (%) (right).

**Figure 4:**
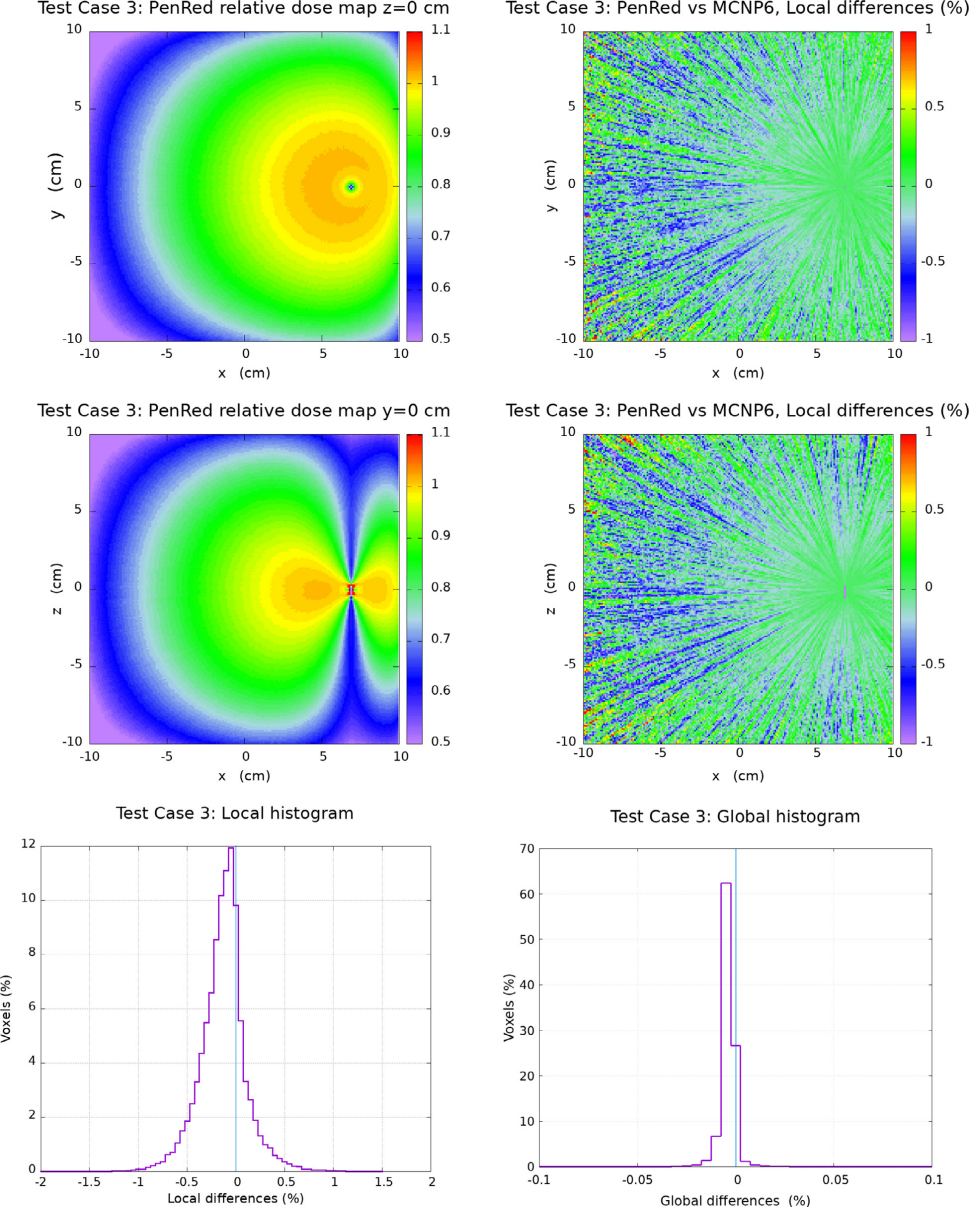
Results for Test Case 3: the “source displaced”, the 20.1 cm side water cube is located in the center of a 51.1 cm side air cube with the generic source displaced 7 cm along the positive *x* axis. Top and middle left images show the PenRed relative dose map for z=0 and y=0 respectively. Top and middle right images show the local differences ratio $\Delta D_{\mathrm{LOCAL}}$ (%) for z=0 and y=0 respectively. Finally, bottom images show the histogram of $\Delta D_{\mathrm{LOCAL}}$ (%) (left) and $\Delta D_{\mathrm{GLOBAL}}$ (%) (right).

**Figure 5:**
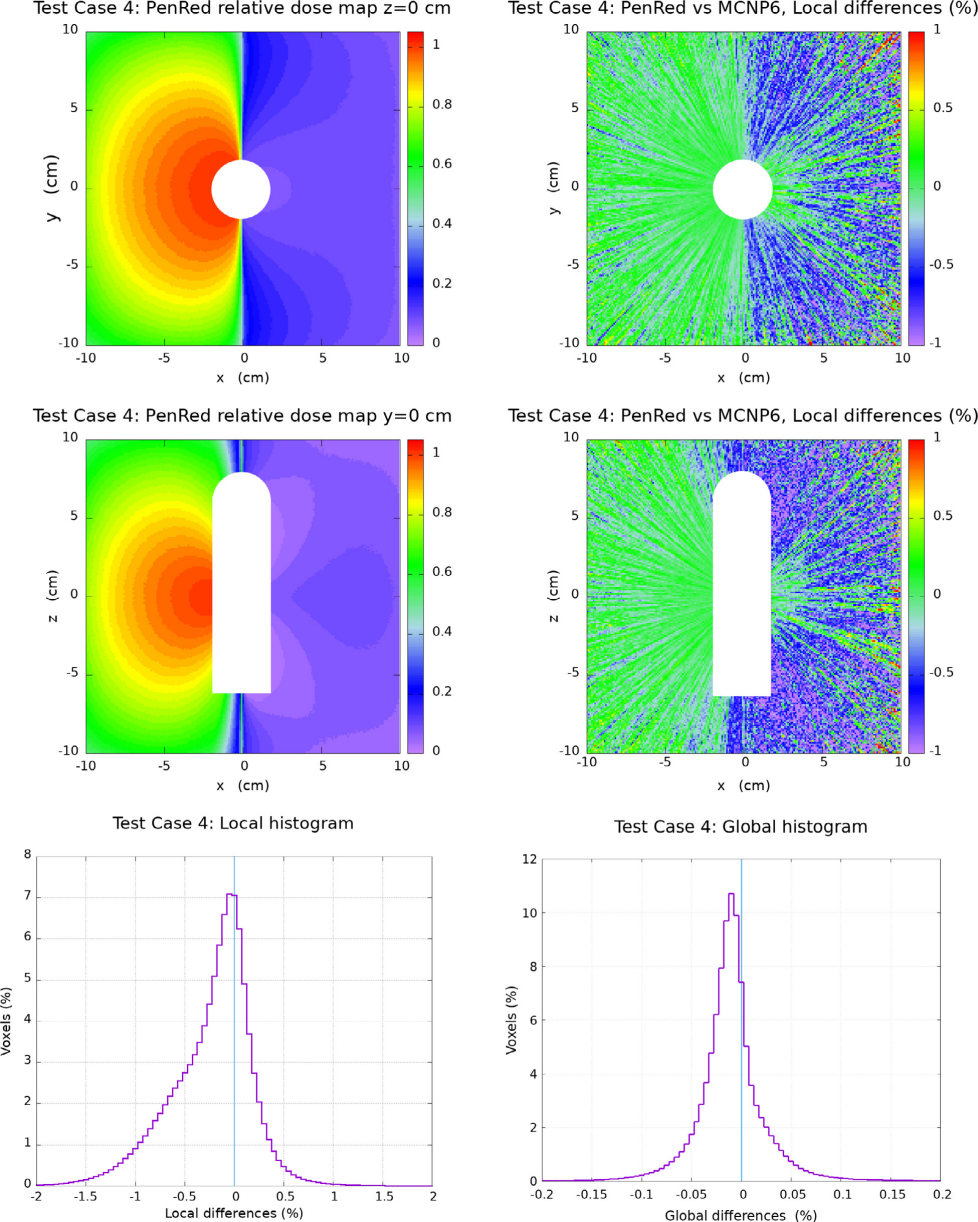
Results for Test Case 4: the “source centered in applicator”, the 20.1 cm side water cube is located in the center of a 51.1 cm side air cube with the generic source centered in the TG-186 shielded applicator. Top and middle left images show the PenRed relative dose map for z=0 and y=0 respectively. Top and middle right images show the local differences ratio $\Delta D_{\mathrm{LOCAL}}$ (%) for z=0 and y=0 respectively. Finally, bottom images show the histogram of $\Delta D_{\mathrm{LOCAL}}$ (%) (left) and $\Delta D_{\mathrm{GLOBAL}}$ (%) (right).

In this section, the results for the water cube phantom tests are presented. The Figure 2, Figure 3, Figure 4, Figure 5 corresponding to each Test Case follow the same organisation. Firstly, top and middle left images show the PenRed relative dose map for the XY and XZ planes, z=0 and y=0, respectively. Secondly, top and middle right images show the local difference ratio $\Delta D_{\mathrm{LOCAL}}$ (%) for z=0 and y=0, respectively. Finally, bottom images show the histogram of $\Delta D_{\mathrm{LOCAL}}$ (%), between −5% and +5% (left) and $\Delta D_{\mathrm{GLOBAL}}$ (%) between −0.4% and +0.4% (right).

With respect to the Test Case 1 analysis, histograms of Fig. 2, show that a total of 99.99% of voxels have a local dose difference ratio within ± 1 %. The maximum of the distribution is at −0.20 % with σ = 0.16 %. Moreover, a total of 99.76% of voxels have a global dose difference ratio within ± 0.1 %, with the maximum of the distribution at −0.005 %, σ = 0.010 %.

For Test Case 2, “source centered in water”, Fig. 3 shows that 99.99% of voxels were also within the ± 1 % for the local dose difference with the maximum of the distribution at −0.15 % with σ = 0.17 %. For the global dose difference ratio, the results show a total of 99.93% of voxels within ± 0.1 %, with the maximum of the distribution at −0.005 % and σ = 0.007 %.

The “source displaced” Test Case, Fig. 4, shows a total of 99.76% of voxels within ± 1 % for the local dose difference with the maximum of the distribution at −0.13 % with σ = 0.24 %. Regarding to the global dose difference ratio, 99.88% of voxels are within the ± 0.1 %, with the maximum of the distribution at −0.004 %, σ = 0.008 %. Since the source is displaced along the *x* positive axis, the left region of the local and global dose difference ratio, shows the highest values. This region is close to the water–air interface and it is the furthest region from the source, resulting in less particle fluence.

Test Case 4 corresponds to the “source centered in applicator” scenario, in which the influence of the shielding has been studied. As it is shown in Fig. 5, for the local dose difference ratio, 94.90% of voxels are within ± 1 %. The distribution is not totally symmetric in this case, showing a tail in the left side of the maximum, which is at −0.23 % with σ = 0.42 %. For the global dose difference ratio distribution the maximum is at −0.010 % with σ = 0.038 %. This histogram shows a total of 97.49% of voxels within ± 0.1 %.

### 3.3 Clinical tests

**Figure 6:**
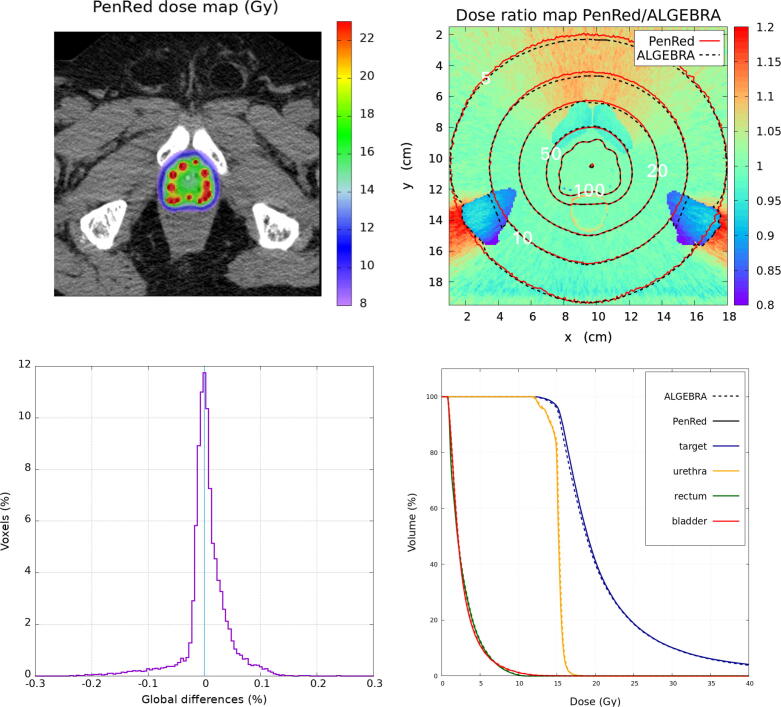
Prostate clinical case with bone structures taken as water. The top-left image shows the PenRed dose distribution. The top-right shows the dose ratio map between PenRed and ALGEBRA results with the isodose lines for both codes. The bottom-left image shows the histogram of $\Delta D_{\mathrm{GLOBAL}}$ (%) ratio and the bottom-right images shows the cumulative dose-volume histogram (DVH) obtained with PenRed for the contoured structures in the CT DICOM of the patient.

**Figure 7:**
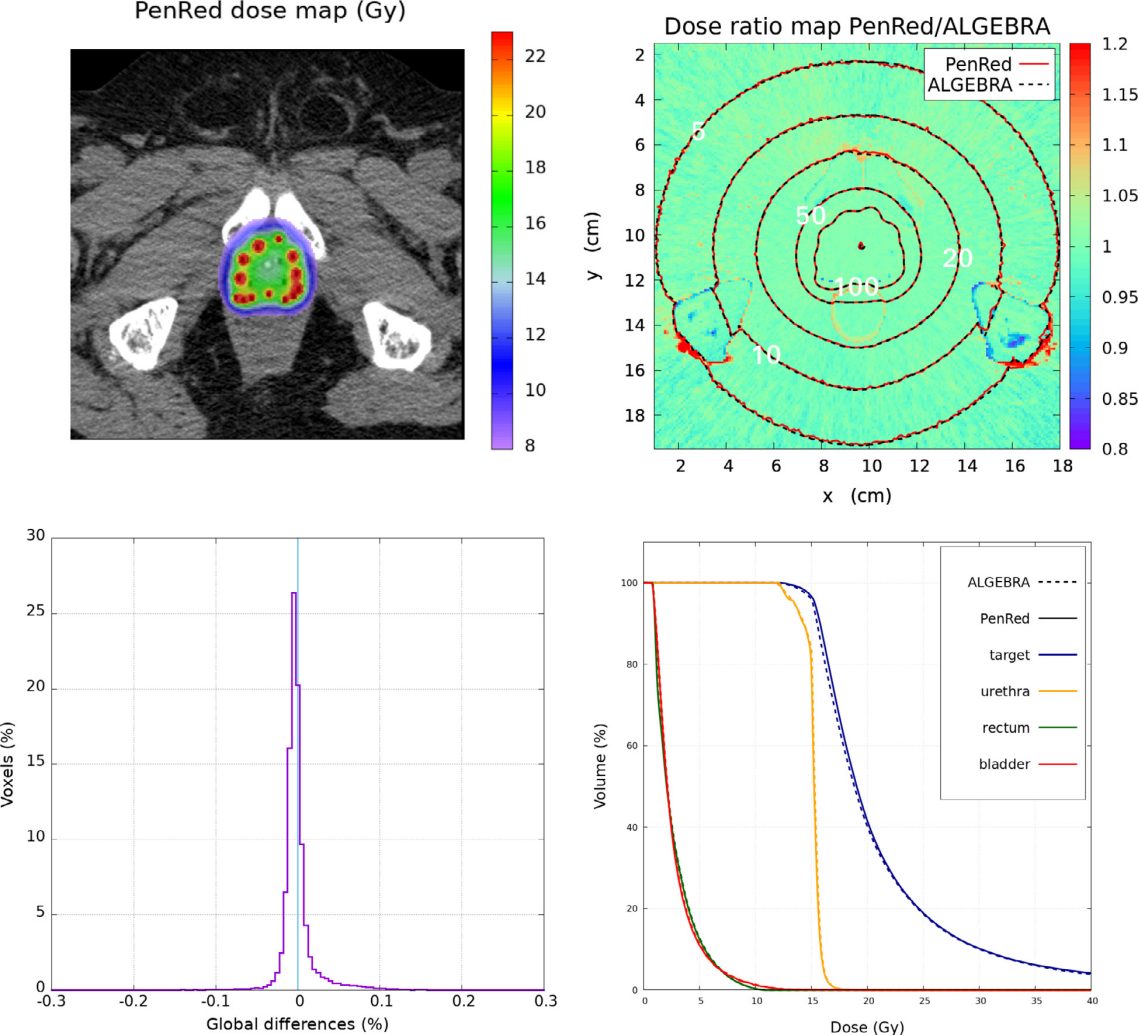
Prostate clinical case with the cortical bone material assignation to the bone structures using intensity range thresholding. The top-left image shows the PenRed dose distribution. The top-right shows the dose ratio map between PenRed and ALGEBRA results with the isodose lines for both codes. The bottom-left image shows the histogram of $\Delta D_{\mathrm{GLOBAL}}$ (%) ratio and the bottom-right images shows the cumulative dose-volume histogram (DVH) obtained with PenRed for the contoured structures in the DICOM-RT files.

**Figure 8:**
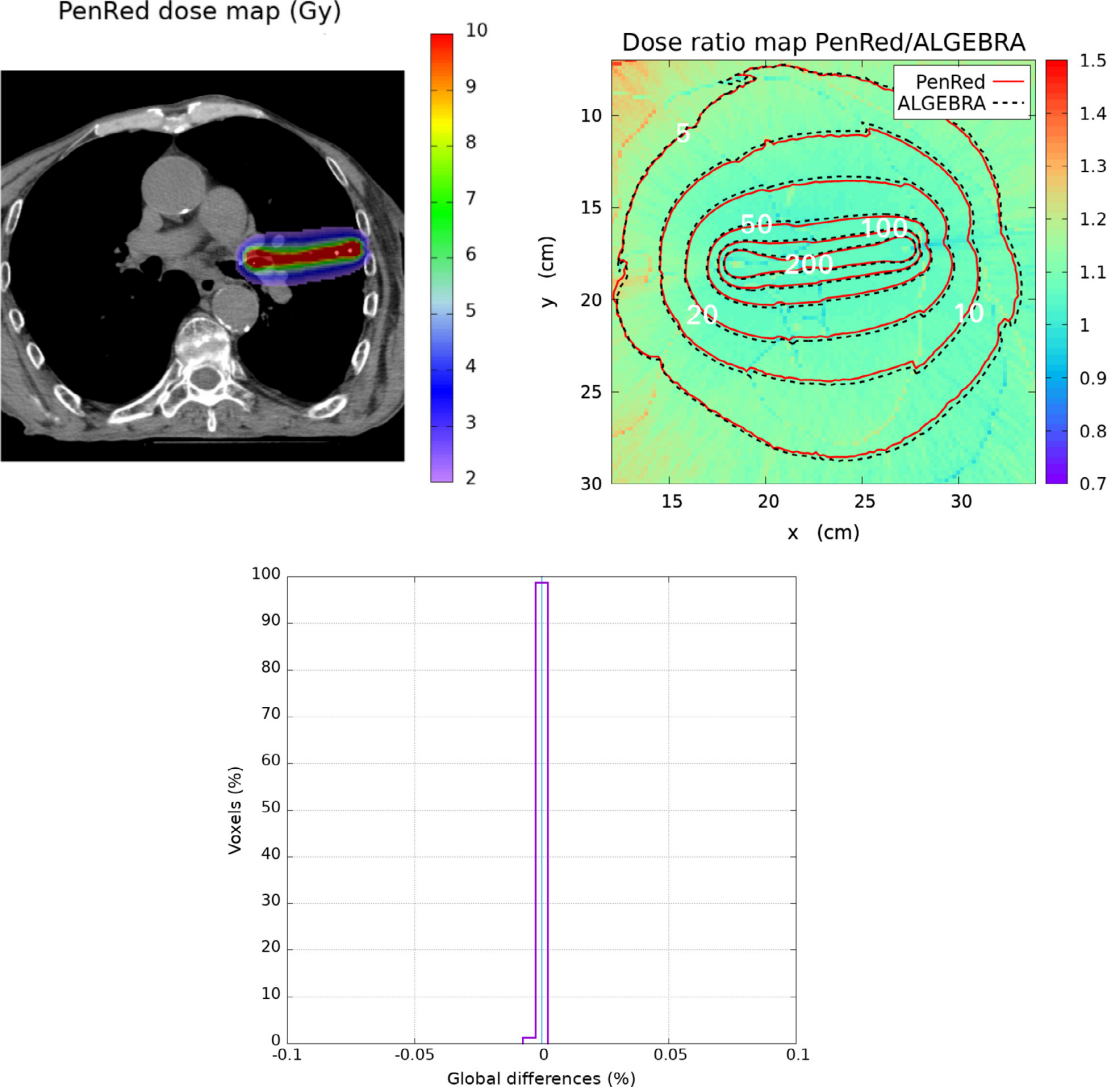
Lung clinical case. The top-left image presents the PenRed dose distribution. The top-right shows the dose ratio map between PenRed and ALGEBRA results with the isodose lines for both codes. The bottom image shows the histogram of $\Delta D_{\mathrm{GLOBAL}}$ (%) ratio.

The prostate case is shown in Figure 6, Figure 7 while the lung case is depicted in Fig. 8. Figures of the prostate case show the dose distribution with PenRed at the XY plane (z = 7.8 cm), the dose ratio map between PenRed and the corresponding reference dataset including the isodose lines for 100%, 50%, 20%, 10% and 5% of the prescribed dose, $\Delta D_{\mathrm{GLOBAL}}$, and the cumulative dose-volume histogram (DVH) obtained with PenRed for the contoured structures reported in the DICOM-RT.

Due to the absence of bone contouring in the prostate case two scenarios were analyzed as described in Section 2.3.2, bone structures taken as water, Fig. 6, or as cortical bone, Fig. 7. The reference dataset assigned cortical bone material to the entire bone structure. DVH metrics for the prostate case are shown in Table 3 and compared with the same metrics obtained with the reference dataset.

**Table 3:** DVH metrics for prostate with and without cortical bone assignation to bone structures

Prostate	Reference	PenRed	PenRed
(Rx = 15 Gy)		(water)	(bone)
$V_{100}$ (%)	96.1	96.6	96.6
$V_{150}$ (%)	27.0	27.0	27.1
$V_{200}$ (%)	10.2	10.2	10.2
D_90_ (Gy)	15.5	15.8	15.8
$D_{50}$ (Gy)	18.9	19.0	19.0
Rectum $D_{2\mathrm{cc}}$ (Gy)	7.6	7.5	7.5

Isodose lines for the bone-as-water prostate case are compatible for 100%, 50%, 20%, 10% and 5% of the prescribed dose. Small deviations can be observed in the bone region. For the global difference histogram, a total of 99.65% of voxels have a global dose difference ratio within ±0.2% with the maximum at −0.003% and σ = 0.040 %. Regarding to the metrics analysis and the comparison with the reference dataset, V_100_ shows a difference of 0.5% while D_90_ and D_50_ show a difference of 0.3 Gy and 0.1 Gy respectively. Rectum $D_{2\mathrm{cc}}$ presents a difference of 0.1 Gy. $V_{150}$ and $V_{200}$ do not present any difference.

When cortical bone material was used to segment the bone structures by intensity range thresholding, the bone region crossed by the isodose lines become closer to the reference isodoses. In this case, 99.80% of the voxels have a global dose difference ratio within the ±0.2% with the maximum at 0.0017% and σ = 0.023 %. Moreover, the DVH metrics present no significant differences when compared with the previous scenario. The $V_{100}$ and $V_{150}$ present a difference with respect to the reference data of 0.5% and 0.1%, respectively. D_90_ and $D_{50}$ show a difference of 0.3 Gy and 0.1 Gy, respectively. Rectum $D_{2\mathrm{cc}}$ presents a difference of 0.1 Gy. $V_{200}$ does not present any difference.

With respect to the lung case both simulations are perfectly clinically compatible, as the mean value of the global dose differences distribution is at $1.1\cdot 10^{-4}$ % with a $\sigma =2.2\cdot 10^{-4}$ % and all the voxels have a global dose difference ratio within ± 0.01%. Small variations in the 10% and 5% isodose can be observed.

## 4 Discussion

### 4.1 WG-DCAB and microSelectron–HDR v2 Ir-192 sources

The reference air-kerma strength for the generic WG-DCAB source was reported in [23] with a value of $(9.801\pm 0.002)\times 10^{-8}$ U Bq^−1^ and dose-rate constant of 1.1109±0.0004 cGy h^−1^ U^−1^ (k=2 Type A uncertainty). Those values were obtained from an ensemble of seven independent MC simulations, reporting values in the range $(9.813-9.784)\times 10^{-8}$ U Bq^−1^ and 1.1114-1.1100 cGy h^−1^ U^−1^. A complete uncertainty analysis lead to a combined Type A and B k=2 uncertainty of about 0.46% for the dose-rate constant and 0.2% for the reference air-kerma strength. The values reported in this work show a good agreement with respect to the reference values, 0.3% for $S_{k}$ and 0.1% for Λ. For the microSelectron–HDR v2 source dose-rate constant, a consensus reference value of 1.109±0.0012 cGy h^−1^ U^−1^ (k=2 Type A uncertainty) has been reported in the 2012 HEBD report [47]. The results obtained agree within 0.09%. The reported agreement is well within the 2007 HEBD report requirements [51], where the MC results and the benchmark data for Λ should be within 2%.

To obtain the complete uncertainty analysis, the recommendations of the TG-43U1, TG-138 [52] and TG-229 reports, have been followed. The Type B uncertainties are estimated taking into account the components shown in Table 4. The uncertainty related to MC code used is assigned following the works [53], [23]. Based on the aforementioned work [53], the uncertainty associated to the phantom composition was set to 0.01 % for both $S_{k}$ and $\overset{˙}{D}$(r = 1 cm, $\theta_{0}$). Then, the Type B uncertainties in (μ/ρ) and $\left(\mu_{\mathrm{en}}/\rho \right)$ are estimated following the study of [54]. Finally, the uncertainties due to the volume averaging are assigned. The Type A uncertainty, due to the tally statistics, is obtained directly from the simulations performed in this work for $S_{k}$ and Λ calculation.

**Table 4:** Uncertainty analysis for the generic HDR ${}^{192}$Ir source.

	$\overset{˙}{D}$(r = 1 cm, $\theta_{0}$)		$S_{k}$	
Component	Type A	Type B	Type A	Type B
Monte-Carlo physics (%)		0.05		0.05
Phantom composition (%)		0.01		0.01
Phantom cross section (μ/ρ)(%)		0.01		0.001
Dose calculation $\left(\mu_{\mathrm{en}}/\rho \right)$ (%)		0.07		0.07
Tally volume averaging (%)		0.20		0.02
Tally statistics (%)	0.01		0.015	
Quadrature sum (%)	0.01	0.22	0.015	0.09
Total uncertainty (%) (k = 1)	0.22		0.09	

It is interesting to note that since the original reference data was published (2012 and 2015) discrepancies beyond expected uncertainties have been reported among different MC results and when comparing with experimental measurements. This has lead to a renew interest in the different possible implementations of low energy cross sections in MC codes, in particular the use of Pratt’s renormalization screening approximation in the description of the photoelectric effect [55], [56].

### 4.2 Water cube phantom tests

Local and Global differences for Test Case 1 are evidence of a perfect agreement with the reference dataset [23], showing a similar performance to other benchmarking calculations in the literature [20]. According to the TG186 classification [12], Test Case 1 is to be understood as a Level 1 commissioning simulation. Such simulations are designed to obtain and benchmark TG-43 dosimetry parameters within the tolerance (2%) recommended by AAPM TG-43U1 report [9]. In this particular case, maximum differences with respect to the reference dataset were reported in the range (+1.6%,-1.4%) depending on the MC code considered [23], being those fully consistent with the results reported here.

As discussed above, Test Case 2 and 3 evaluate the relevance of the lack of scattering material in the vicinity of the source. Test Case 2 is designed to study the influence of a smaller water phantom as compared to the one recommended by TG-43 while Test Case 3 presents a more extreme case where the source is located close to a water–air interface. In Ma et al. [24] the $\Delta D_{\mathrm{Local}}$ and $\Delta D_{\mathrm{Global}}$ range values where 95% of the voxels are located was discussed, being in the case of the local differences between ±0.4% to ±0.8% for Test Case 2 and ±0.6% to ±1.1% for Test Case 3 depending on the MC code considered. Systematic deviations of the $\Delta D_{\mathrm{Local}}$ distributions were reported in the range ±0.1% with standard deviations in the range (0.21%-0.41%) (Test Case 2) and (-0.06% to +0.12%) with standard deviations in the range (0.31%-0.54%) (Test Case 3). These results are in agreement with those obtained with PenRed, where more than 99.8% of voxels are reported within ± 1 % for local dose differences. A similar good agreement as compared with the reference data and other MC codes is observed for the case of the global dose differences.

Test Case 4 is particularly interesting because it not only incorporates the lack of scattering but also the presence of a high *Z* shield in the simulations, hence dividing the phantom in shielded and unshielded volumes. This asymmetry is reflected in the $\Delta D_{\mathrm{Local}}$, and to a lower extend also in the $\Delta D_{\mathrm{Global}}$, distributions. In Ma et al. [24] a systematic $\Delta D_{\mathrm{Local}}$ deviation of about 0.5%-1% with respect to the reference dataset for those voxels located in the shielded part of the phantom (see Figure 4, Figure 5 of Ref. [24]) was observed for all the MC codes discussed (egs_brachy, ALGEBRA, Geant4, BrachyDose, and Penelope). This effect can be also observed in the PenRed datasets, see Fig. 5, although attenuated. In spite of that, there is general good agreement with the reference dataset, 94.90% of $\Delta D_{\mathrm{Local}}$ voxels are within ± 1 %, that compares perfectly with typical agreements of other MC codes, where 95% of $\Delta D_{\mathrm{Local}}$ where typically within ± 2–3 %. $\Delta D_{\mathrm{Global}}$ shown a better agreement than other codes, where 95% of the voxels are located within ±0.04% while for PenRed 97.49% of voxels are within ± 0.1 %. Ma et al. [24] proposed that: (1) Type A uncertainties are larger in the shielded area due to the reduced statistics and (2) the fact that the photon spectrum reaching the shielded region differs greatly from the one radiating into the unshielded parts of the phantom might require larger Type B uncertainties due to differences in the photon spectra and cross-section data.

All four cases simulated with PenRed show excellent agreement within Type A and B uncertainties with the reference data, in some cases even better than other benchmarking calculations reported in the literature.

### 4.3 Clinical tests

The existence of differences due to the material segmentation in the bone structures in the prostate case is restricted to the bone itself and to the low-dose regions leeward behind the bone, see Figure 6, Figure 7. Such minor differences do not affect the target or the organs at risk and therefore they have no clinical significance. There is a much large number of possible sources of differences in a clinical case than in the WG-DCAB test cases discussed above. Therefore, the preferred tool for benchmarking MC clinical simulations is the comparison of $\Delta D_{\mathrm{Global}}$ distributions and dose-volume histograms. In the clinical prostate case more than 99.8% of the voxels present $\Delta D_{\mathrm{Global}}$ values within ±0.2%.

Typical DVH indexes for prostate include $V_{100/150/200}$ and $D_{90}$ of the target, $D_{2\mathrm{cc}}$ for the rectum and $D_{10}$ and $D_{30}$ for the urethra. Recommended values for BT monotherapy being $V_{100}>95\%,V_{150}<40\%,D_{90}>100\%$, larger than 15 Gy in this particular case, and $D_{2\mathrm{cc}}<10$ Gy [57]. Therefore, the minor differences observed in the DVH indexes summarized in Table 3 and in the DVH curves (Fig. 7) have a negligible impact on a clinical case. Thus, emphasizing the perfect agreement of PenRed with the reference dataset.

In the palliative lung case, Fig. 8, only small differences, less than 1 mm displacement, were observed in some regions of the low dose isodoses. Such values, together with the fact that no voxel presented global dose difference ratios larger than 0.01% proved that any minor difference reported with respect to the reference dataset would not be clinically significant.

## 5 Conclusions

The open-access MC code PenRed has been validated for HDR brachytherapy. PenRed incorporates all the physical libraries designed for its use in the original Penelope package together with a modular and flexible structure using an Object-Oriented programming model. New tallies and sources, specifically tailored for its use in brachytherapy, have been included in the PenRed distribution. This is a fully parallel MC code which implements parallelism in two ways: using standard C++ threads for shared memory and through MPI standard for distributed memory. It also allows the user to incorporate all the information contained in a clinical DICOM structure into a MC simulation in a simple and straightforward way. Six different Test Cases have been discussed. Overall, all simulations showed an excellent agreement with the reference data. First, the four commissioning tests cases designed by the AAPM/ESTRO/ABG WG-DCAB were explored, showing local agreements better than the expected Type A uncertainties in excess of 99.8% of the voxels for Tests Cases 1 to 3 and 94.9% for Test Case 4. The comparison for global difference offered an even better picture, where more than 99.8% and 95.5% of the voxels present differences less than 0.1% of the reference dose for Test Cases 1 to 3 and 4 respectively. The two clinical cases analyzed presented global differences smaller than 0.2% in 99.8% of the voxels and negligible differences in the dose-volume histogram indexes. These results, together with the new source and tallies incorporated into the distribution, makes evident that PenRed can be considered a reliable, precise, and easy-to-use MC code for brachytherapy simulations.

## Declaration of Competing Interest

The authors declare that they have no known competing financial interests or personal relationships that could have appeared to influence the work reported in this paper.

## Footnotes

1 https://github.com/PenRed/PenRed.

2 https://github.com/PenRed/PenRed.
